# Efficacy and Safety of NaoShuanTong Capsule in the Treatment of Ischemic Stroke: A Meta-Analysis

**DOI:** 10.3389/fphar.2019.01133

**Published:** 2019-10-11

**Authors:** Hanlai Zhang, Yanwei Xing, Jingling Chang, Liqin Wang, Na An, Chao Tian, Mengchen Yuan, Xinyu Yang, Hongcai Shang, Ying Gao, Yonghong Gao

**Affiliations:** ^1^Key Laboratory of Chinese Internal Medicine of Ministry of Education and Beijing, Dongzhimen Hospital Affiliated to Beijing University of Chinese Medicine, Beijing, China; ^2^Guang’an Men Hospital, China Academy of Chinese Medical Sciences, Beijing, China; ^3^Chinese Medicine Research Room of Encephalopathy Syndrome and Treatment of the State Administration of TCM People's Republic of China, Dongzhimen Hospital Affiliated to Beijing University of Chinese Medicine, Beijing, China

**Keywords:** NaoShuanTong capsule, ischemic stroke, cerebral infarction, randomized controlled trial, meta-analysis, systematic review

## Abstract

**Background:** Ischemic stroke (IS) is a leading cause of death and long-term disability worldwide. The NaoShuanTong capsule (NSTC), a traditional Chinese patent medicine, has been extensively used in the treatment of stroke in China. However, the clinical efficacy and safety of this treatment has not been statistically and systematically verified by any comprehensive pooled analysis. We therefore performed a meta-analysis to evaluate the efficacy and safety of NSTC in the treatment of IS.

**Methods:** Randomized controlled trials (RCTs) of NSTC in the treatment of IS conducted before September 2018 were retrieved from five databases, according to specific inclusion and exclusion criteria. Two investigators independently reviewed the included studies and extracted relevant data. The methodological quality of the included studies was assessed using criteria from the Cochrane Handbook, and analyzed using Review Manager 5.3 software.

**Results:** Thirteen RCTs comprising a total of 1,360 participants were included in this study. NSTC was shown to significantly improve the overall response rate (*OR* = 3.04, 95% CI [1.76, 5.26], *P* < 0.00001), and neurological function (NSTC increased Modified Barthel Index (*MD* = 8.15, 95% CI [3.79, 12.52], *P* = 0.0005), Functional Independence Measure (*MD* = 29.61, 95% CI [10.11, 49.10], *P* = 0.003) and European Stroke Scale scores (*MD* = 8.51, 95% CI [7.00, 10.01], *P* = 0.03). In addition, NSTC significantly increased serum adiponectin level (*MD* = 0.66, 95% CI [0.23, 1.08], *P* = 0.002). Moreover, NSTC reduced atherosclerotic plaque area (*MD* = -2.24, 95% CI [-4.02, -0.46], *P* = 0.01) and intima-media thickness (*MD* = -0.09, 95% CI [-0.13, -0.05], *P* < 0.0001). However, there was no significant difference between NSTC treatment and conventional therapy with respect to Fugl-Meyer Assessment score (*MD* = 10.59, 95% CI [-1.78, 22.96], *P* = 0.09) or Crouse score (*MD* = -0.78, 95% CI [-1.79, -0.22], *P* = 0.13).

**Conclusions:** The results of this meta-analysis showed that NSTC exhibits efficacy in the treatment of cerebral infarction. NSTC can improve the overall response rate and neurological function, increase blood adiponectin, reduce neurological deficits, and decrease atherosclerotic plaque area.

## Introduction

Ischemic stroke (IS) is a leading cause of mortality and disability worldwide, responsible for 5.5 million deaths and 116.4 million disability-adjusted life-years in 2016 (Collaborators, 2019). In addition, stroke imposes significant psychological pressure and a substantial economic burden on patients (Hay et al., 2017). Ischemic stroke is the major manifestation of stroke, occurring in (87%) of stroke cases (Radu et al., 2017), and usually occurs due to the occlusion of an artery, notably as a result of atherosclerotic disease of the middle cerebral artery (Wong et al., 2016). The ischemic cascade starts with a reduction in blood flow and energy supply, which subsequently leads to excitotoxicity, oxidative stress, and cell death (Sifat et al., 2017; Venkat et al., 2017). Typically, this causes serious damage to the central nervous system (Santos Samary et al., 2016). Currently, the foremost therapeutic regimen employed for ischemic stroke includes administration of an antiplatelet agent, fibrinolytic therapy, and endovascular treatment (Yan et al., 2015; Linfante and Cipolla, 2016; Venkat et al., 2018). Unfortunately, however, the “time window” and risk of bleeding of this regimen limit its clinical application (Li et al., 2019).

NaoShuanTong capsule (NSTC) is a Chinese patent medicine which has been approved by the State Food and Drug Administration for the treatment of ischemic stroke and hemorrhage. NSTC contains five ingredients, namely, *Pollen Typhae* (the dry pollen of *Typha angustifolia* L), *Radix Paeoniae Rubra* (the root of *Paeoniae Radix Rubra*), Radix Curcumae (the root of *Curcuma wenyujin* Y. H. Chen et C. Ling), *Rhizoma Gastrodiae* (the root of *Gastrodia elata* B1), and Radix Rhapontici (the radix of *Rhaponticum uniflorum (L.) DC*) (Huang et al., 2015). According to the high-performance liquid chromatography analysis (**Supplemental Figure 1**), the currently known chemical components of NSTC mainly include: paeoniﬂorin, ecdysterone, typhaneoside, isorhamnetin-3-O-neohesperidoside and so on (Chen Si and Liu, 2013; Liu et al., 2014). Studies have shown that NSTC can alleviate hemorheological disorders, improve cerebral energy metabolism in animal models, and speed up the recovery of nerve function by reducing the apoptosis of neurons and astrocytes caused by ischemia, protecting the neuro vascular unit from cerebral infarction and its associated repercussions, and reducing brain tissue injury by protecting hemorheology and cerebral energy metabolism disorders in pharmacology studies (Shi et al., 2012; Liu et al., 2014). Clinical studies have also indicated that NSTC has positive clinical efficacy and safety in extending survival after stroke, improving neurological function, reducing neurological deficits, lowering blood fat, and shrinking atherosclerotic plaques (Ye et al., 2015; Liang et al., 2017).

Although a number of clinical studies have indicated that NSTC is beneficial in the treatment of stroke, there currently exists no comprehensive and systematic evidence to confirm its clinical efficacy and safety. Therefore, we performed this meta-analysis to evaluate the efficacy of NSTC in the treatment of ischemic stroke.

## Methods

### Search Strategy and Literature Selection

This meta-analysis followed the Preferred Reporting Items for Systematic Reviews and Meta-Analyses (PRISMA) statement (Hutton et al., 2015). No pre-specified protocol was schemed for this analysis. A comprehensive search of relevant articles was conducted independently by two investigators using five bibliographical databases, namely, China National Knowledge Internet (CNKI): http://www.cnki.net, Cochrane Library: https://www.cochranelibrary.com, China Science and Technology Journal Database (VIP): http://www.cqvip.com, PubMed: https://www.ncbi.nlm.nih.gov, and Wanfang Database: http://www.wanfangdata.com.cn/index.html. The search was performed in September 2018 and no lower date limit was applied. Combinatorial keywords were utilized for this search: “Naoshuantong Capsule OR NST Capsule OR NSTC”, “ischemic stroke OR cerebral ischemia OR cerebral infarction OR stroke”, and “clinical randomized controlled trial”. Subsequently, we retrieved additional publications using manual retrieval of references from recent reviews and relevant original studies. Only full-text references published in peer-reviewed journals in English or Chinese were included. Any disagreements were solved by the two researchers reaching a consensus or by a third party.

### Inclusion and Exclusion Criteria

Studies were included if they met the following conditions: 1) Participants: patients diagnosed with IS by speciﬁc criteria; 2) Intervention: the experimental group comprised patients treated with NSTC alone or combined with conventional therapy; 3) Comparator: the control group comprised patients treated with a placebo or conventional treatment; 4) Outcome: comprised information of defined events which could be examined independently of other intervention components, such as odds ratio (OR), risk ratio (RR), hazard ratio (HR), or necessary raw data with 95% confidence intervals (CIs); 5) Study design: randomized controlled trial (RCT); 6) NSTC was provided by Guangdong Huanan Pharmaceutical Co., Ltd (Guangdong, China).

Studies were excluded due to: 1) the inclusion of acutely ill subjects; 2) unsuitable outcomes for meta-analysis; 3) duplicate publications (only the first publication was included); 4) unpublished literatures; 5) case reports, reviews, animal experiments, conference abstracts, letters, and expert opinions; 6) publication in a language other than English or Chinese.

### Data Extraction and Quality Assessment

The following information was extracted from each RCT independently by two investigators: name of the first author, publication year, study period, demographic and health characteristics, sample size, intervention, outcomes, and adverse events.

The methodological quality of all the included RCTs was assessed using the Cochrane Handbook for Systematic Reviews of Interventions (Higgins et al., 2003). This was conducted systematically and comprehensively using seven parameters, namely sequence generation, allocation concealment, blinding of participants and personnel, blinding of outcome assessors, incomplete outcome data, selective outcome reporting, and other sources of bias. The quality of each item was assessed using three levels of bias - “low risk,” “high risk,” or “unclear risk”, and graphs depicting this information were then created. When more than ten articles were included in the analysis, a funnel plot would be utilized to evaluate publication bias.

### Statistical Analysis

Review Manager, version 5.3 software (Revman; The Cochrane Collaboration, Oxford, U.K.) was used for statistical analyses. Effect estimates were extracted, such as OR, RR, and raw data, and were presented as the mean difference (MD) or OR and 95% CIs. Heterogeneity was assessed using the *Q* (qualitative) and *I* ^2^ statistic tests (quantitative). The statistical model employed was determined by the results of the heterogeneity test in each study. When *P* ≥ 0.1 and *I*^2^ < 50%, this indicated that there was no or low heterogeneity between the study materials, and a fixed effects model was used for the analysis. In contrast, when *P* < 0.1 and *I*^2^ > 50%, this indicated significant heterogeneity between the research data, and thus a random effects model was adopted for the analysis. When the results of the two heterogeneity tests were inconsistent, *I*^2^ was used as the main evaluation method.

## Results

### Description of Included Trials

**Figure 1:**
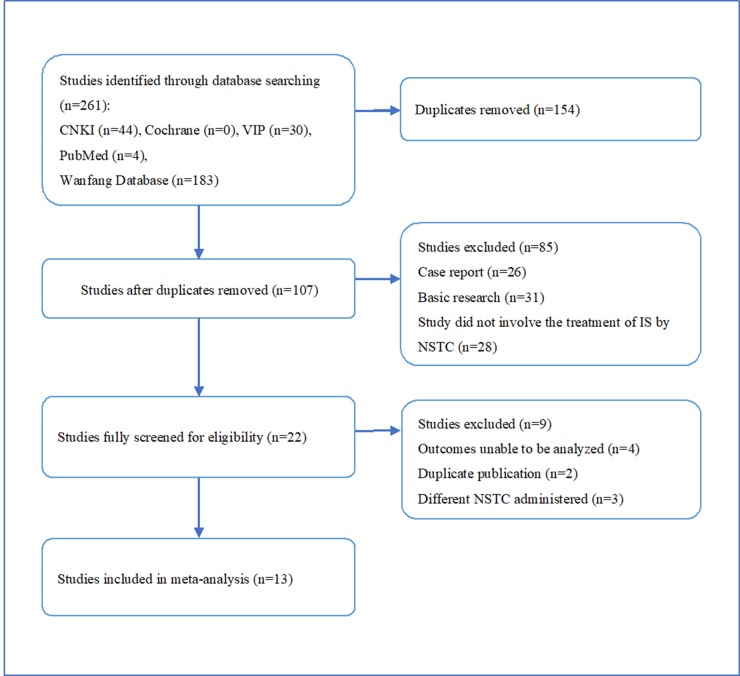
Flow diagram depicting the process of the literature screening.

We identified 261 potentially relevant articles from five databases, comprising 44 papers from CNKI, 183 from Wan Fang, 30 from VIP, 4 from PubMed, and none from Cochrane Library. After the removal of duplicates, 107 articles remained. After assessment of the titles and abstracts, 85 papers were excluded for at least one of following reasons: (1) it was a case report or review; (2) it included animal research; (3) it did not comprise research about stroke or ischemia treated with NSTC. The full text of the remaining 22 articles reporting the clinical efficacy of NSTC in the treatment of ischemic stroke was then read. Four studies were excluded because the outcomes could not be analyzed, two because of duplicate publication, and three because they did not use the aforementioned NSTC. Ultimately, thirteen eligible studies were utilized for the meta-analysis. This process is depicted in **Figure 1**.

A total of 1,360 participants were included (673 in the experimental group and 687 in the control group). The sample sizes of the included studies ranged from 44 to 200. The duration of treatment ranged from four weeks to twelve months. The dosage of NSTC administered was two pills three times a day in one trial, and three pills three times a day in the other trials. The experimental group was treated with NSTC combined with antiplatelet drugs in five trials, NSTC combined with aspirin and statin in five trials, NSTC combined with conventional Chinese medicinal products (ShuXueTong injection) in one trial, and NSTC as monotherapy in one trial. The control group was treated with antiplatelet drugs in four trials, aspirin and statins in seven trails, and Chinese herb medicine monotherapy (ShuXueTong injection and NaoXinTong capsule) in two trials.

### Quality Assessment of Selected Studies

**Figure 2:**
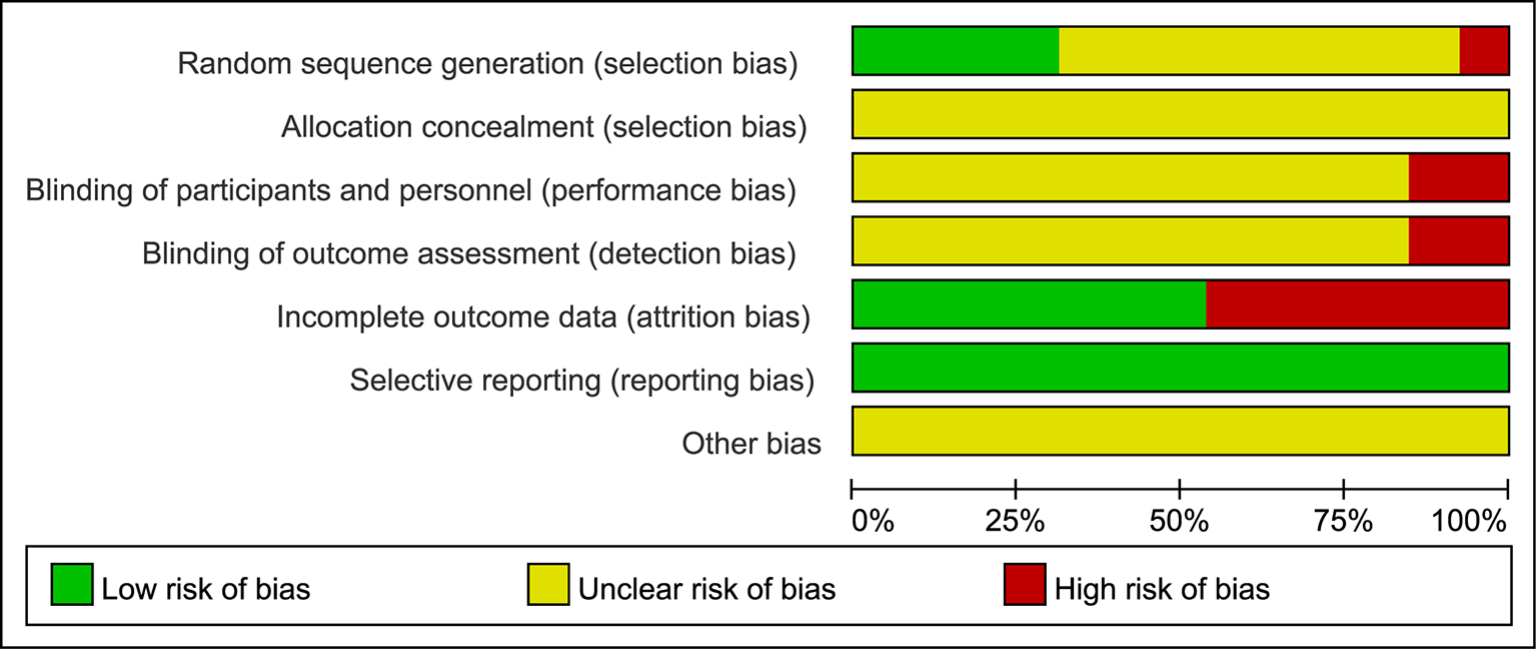
Risk of bias graph. The judgments of the reviewing authors about each domain of bias are presented as percentages of all included studies. The quality of the selected studies was assessed according to the Cochrane criteria.

**Figure 3:**
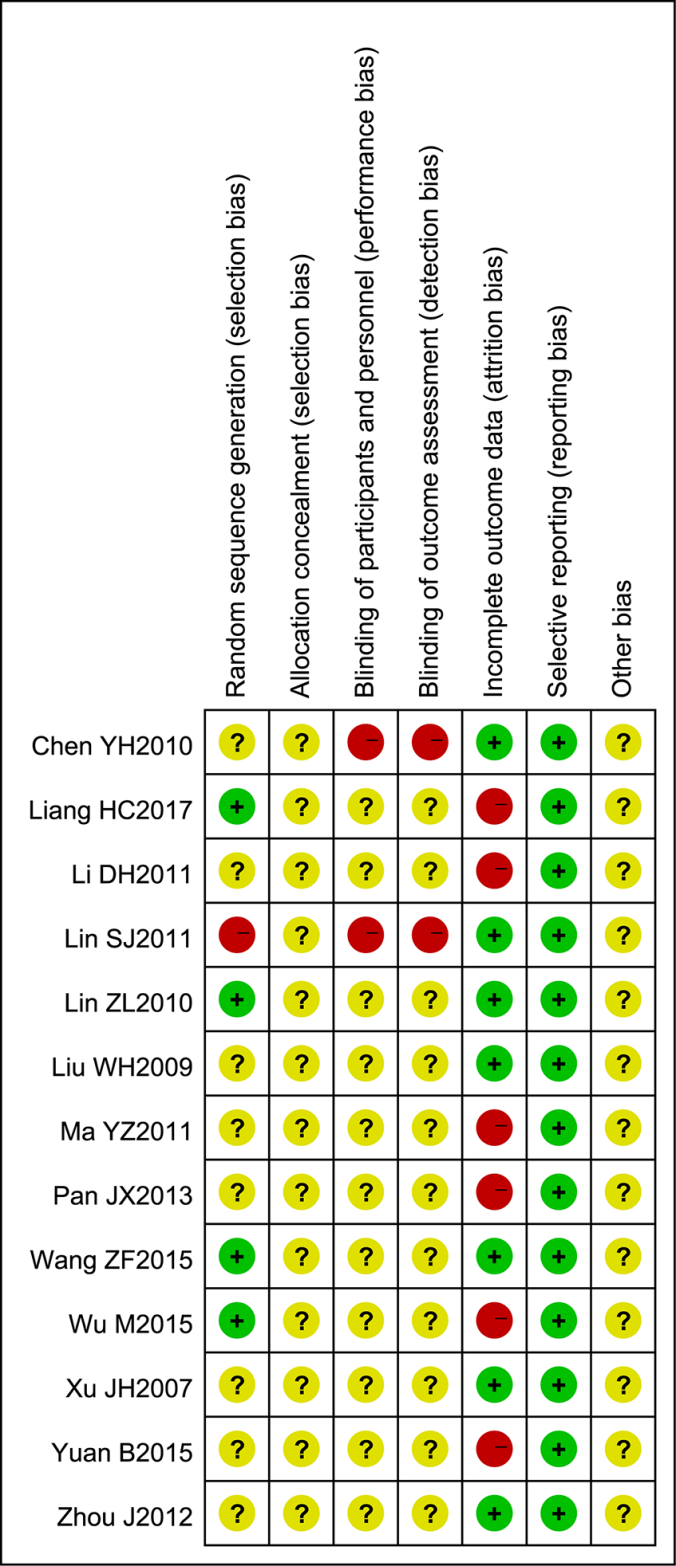
Risk of bias summary. The judgments of the reviewing authors about each domain of bias for each included study are summarized.

Although all trials claimed to be RCTs, only four reported appropriate methods of random sequence generation. In addition, one trial used an inappropriate method of randomization. Allocation concealment was not mentioned in all trials. Two trials reported blinding - one was double blind and the other single blind - but neither described the method of blinding. Twenty patients withdrew or dropped out of five trials. There were no signiﬁcant differences in baseline data between the trials (**Table 1**). The sample size of all trials was small and none of the included trials provided a pretrial estimation of sample size. The results of the bias risk assessment are presented in **Figures 2** and **3**.

**Table 1:** Characteristics of the included studies.

Study ID	Intervention		Sample size [age (median (IQR)/mean ± SD)]		Baseline comparison	Treatment course	Outcomes	Adverse event
	Experimental	Control	Experimental	Control				
Li DH2011	NSTC 2# tid+Aspirin	Aspirin	33(46-78)	33(46-78)	Y	1M	MBI, FIM, CSS	NM
Lin ZL2010	NSTC 3# tid+Aspirin	Aspirin	22(65.96 ± 4.78)	23(66.83 ± 4.65)	Y	1M	CSS, FIM, MBI	N
Wang ZF2015	NSTC 3# tid+Clopidogrel	Clopidogrel	21(70.14 ± 9.28)	24(69.04 ± 8.7)	Y	4W	FMA, MBI	NM
Ma YZ2011	NSTC 3# tid+Aspirin+Citicoline	Aspirin+Citicoline	35(59.8 ± 8.3)	30(60.1 ± 7.2)	Y	28D	OR, FMA, MBI	NM
Lin SJ2011	NSTC 3# tid+Aspirin	Simvastatin+Aspirin	48(50.18 ± 6.89)	44(51.28 ± 7.09)	Y	12M	TG, TC, LDL, HDL, IMT, CS, AAP	Y
Zhou J2012	NSTC 3# tid+Aspirin+Atorvastatin	Aspirin+Atorvastatin	66(63.4 ± 8.7)	66(63.4 ± 8.7)	Y	6M	IMT, AAPA, CS	N
Wu M2015	NSTC 3# tid+Aspirin+Atorvastatin	Aspirin+Atorvastatin	45(45-75)	44(45-75)	Y	4W	ESS, FIM	NM
Pan JX2013	NSTC 3# tid+Aspirin+Atorvastatin	Aspirin+Atorvastatin	66(63.5 ± 15.9)	69(62.5 ± 16.7)	Y	4W	OR, ESS, MBI	NM
Yuan B201 5	NSTC 3# tid+Aspirin+Breviscapine 50mg in 5%GS ivgtt	Atorvastatin+Aspirin+Breviscapine 50mg in 5%GS ivgtt	25(47.0 ± 8.6)	25(49.2 ± 10.7)	Y	28D	APN	NM
Liang HC2017	NSTC 3# tid+Aspirin+Atorvastatin	Aspirin+Atorvastatin	58(62.17 ± 6.49)	58(62.56 ± 6.55)	Y	6M	IMT, AAPA, CS, APN	NM
Chen YH2010	NSTC 3# tid+Aspirin+Atorvastatin	Aspirin+Atorvastatin	60(67.78 ± 19.34)	65(65.97 ± 17.76)	Y	3M	OR, MBI, TG, TC, LDL, HDL	N
Liu WH2009	NSTC 3# tid+ ShuXueTong injection 6ml in NS/GS250ml ivgtt qd	ShuXueTong injection 6ml in NS/GS250ml ivgtt qd	100(60.23 ± 14.32)	100(63.16 ± 13.05)	Y	4W	OR, ESS	NM
Xu JH2007	NSTC 3# tid	NaoXinTong capsule 3# tid	52(65.3 ± 10.78)	60(65.3 ± 10.78)	Y	3M	NIHSS, MBI	NM

AAP, area of atherosclerotic plaque; APN, adiponectin; CS, the Crouse score; CSS, the Chinese Stroke Scale; ESS, the European Stroke Scale; FMA, the Fugl-Meyer Assessment; FIM, the Functional Independence Measure scale; HDL, high density lipoprotein; IMT, intima-media thickness; IS, ischemic stroke; LDL, low density lipoprotein; MBI, the Modified Barthel Index; NIHSS, National Institute of Health Stroke Scale; NSTC, NaoShuanTong capsule; OR, overall response; TC, triglycerides; TG, total cholesterol.

### Effects of the Intervention

#### Overall Response Rate of NSTC

**Figure 4:**
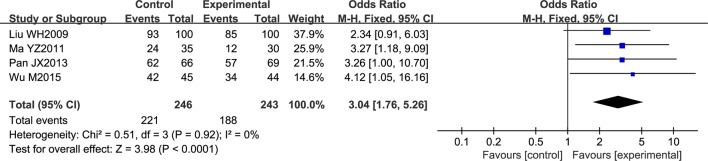
Forest plot of the overall response rate in ischemic stroke patients treated with NSTC and conventional therapy (experimental group) and conventional therapy alone (control group).

Four trials contributed to this analysis, comprising a total of 489 patients. A fixed effects model was used for analysis according to heterogeneity testing (P = 0.92; I2 = 0%). Over the treatment period, the overall response rate was higher in the experimental group than in control group (OR = 3.04; 95% CI [1.76, 5.26]; *P* < 0.0001) (**Figure 4**).

#### Neurological Function Score

**Figure 5:**
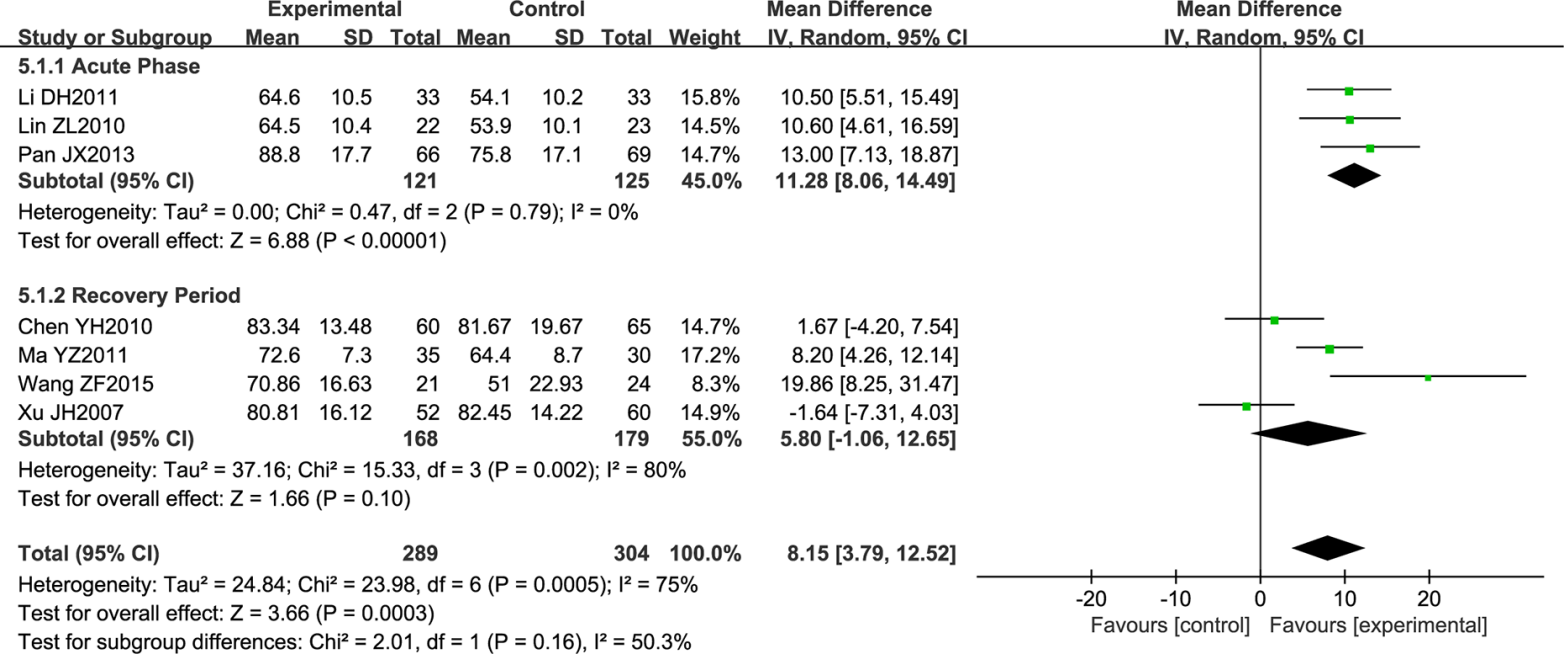
Forest plot of Modified Barthel Index score in ischemic stroke patients treated with NSTC and conventional therapy (experimental group) and conventional therapy alone (control group).

Seven trials comprising a total of 593 patients measured neurological function using the Modified Barthel Index (MBI). Due to the high heterogeneity noted, subgroup analysis was performed according to the stage of disease. However, the heterogeneity remained high (*P* = 0.79; *I*2 = 0%, *P* = 0.002; *I*2 = 80%), so a random effects model was used for analysis. NSTC was found to improve neurological function in acute stroke patients (MD = 11.28; 95% CI [8.06, 14.49]; *P* < 0.00001). However, NSTC did not have a statistically significant effect on patients in the recovery period (as demonstrated by the diamond intersecting the equivalent line) (**Figure 5**). Therefore, we speculate that the main source of heterogeneity is the stage of disease.

**Figure 6:**
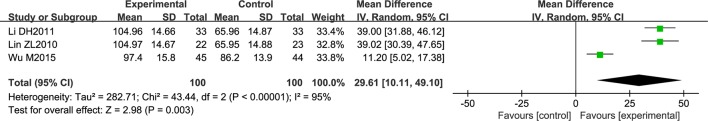
Forest plot of Functional Independence Measure score in ischemic stroke patients treated with NSTC and conventional therapy (experimental group) and conventional therapy alone (control group).

Three studies comprising 200 patients measured neurological function by the Functional Independence Measure scale (FIM). All included patients were in the acute phase of the disease. A random effects model was used for the statistical analysis due to the heterogeneity noted (*P* < 0.00001; *I^2^* = 95%). NSTC was found to improve neurological function (MD = 29.61; 95% CI [10.11, 49.10]; *P* = 0.003) (**Figure 6**). In order to explore the source of heterogeneity, We conducted a sensitivity analysis by sequentially omitting each employed study. We found that the study by Wu M 2015 substantially influenced the pooled MD. Removing this study yielded a pronounced improvement in FIM (MD = 39.01; 95% CI [33.51, 44.50]; *P* < 0.00003) with low heterogeneity (*P* = 0.100; *I^2^* = 0%).

**Figure 7:**
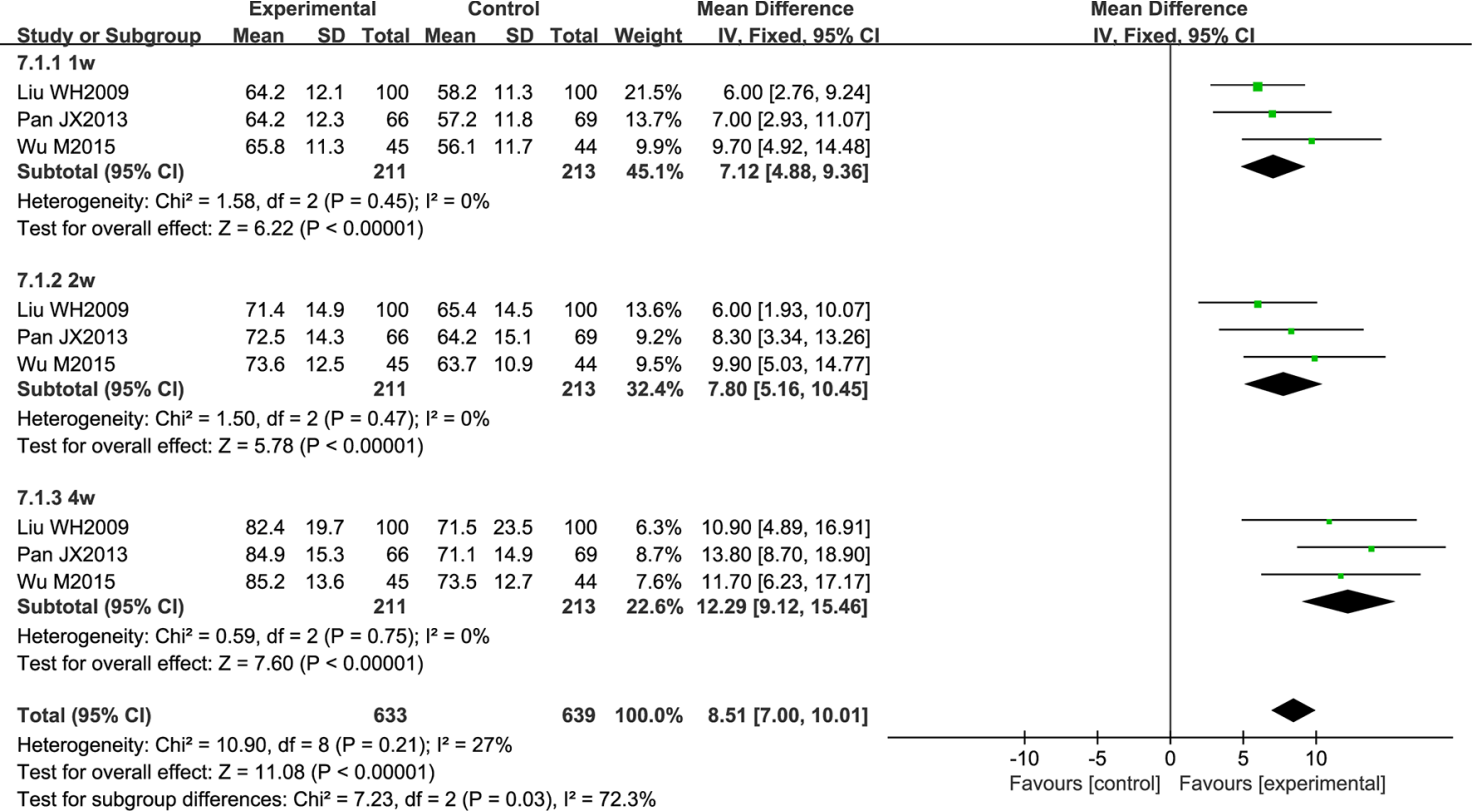
Forest plot of European Stroke Scale score in ischemic stroke patients treated with NSTC and conventional therapy (experimental group) and conventional therapy alone (control group).

Three studies reported an improvement in neurological function, evaluated using the European Stroke Scale (ESS). All patients had acute cerebral infarction. The data were analyzed using a fixed effects model according to the heterogeneity test (*P* = 0.21; *I^2^* = 27%). Over the treatment period, the total effective rate was higher in the experimental group than in the control group (MD = 8.51; 95% CI [7.00, 10.01]; *P* < 0.00001). A subgroup analysis was conducted according to the period of treatment, and the results showed that the curative effect was proportional to the course of treatment (MD = 7.12; 95% CI [4.88, 9.36]; *P* < 0.00001, MD = 7.80; 95% CI [5.16, 10.45]; *P* < 0.00001, MD = 12.29; 95% CI [9.12, 15.46]; *P* < 0.00001) (**Figure 7**).

**Figure 8:**
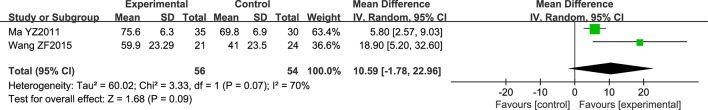
Forest plot of Fugl-Meyer Assessment score in ischemic stroke patients treated with NSTC and conventional therapy (experimental group) and conventional therapy alone (control group).

Two studies comprising a total of 109 patients employed the Fugl-Meyer assessment (FMA). All patients had acute cerebral infarction. The data were analyzed using a random effects model according to the heterogeneity test (*P* = 0.07; *I^2^* = 70%). The obtained results were not statistically significant (the diamond intersects the equivalent line) (**Figure 8**).

#### Blood Lipid Level and Atheromatous Plaque

**Figure 9:**
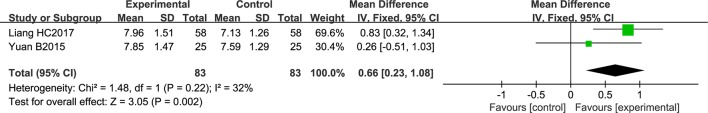
Forest plot of adiponectin level in ischemic stroke patients treated with NSTC and conventional therapy (experimental group) and conventional therapy alone (control group).

Two studies comprising a total of 166 patients reported adiponectin level. No significant heterogeneity was noted among the individual trials (*P* = 0.22; *I* ^2^ = 32%). Using a fixed effects model, NSTC was found to significantly increase adiponectin level (MD = 0.66; 95% CI [0.23, 1.08]; *P* = 0.002) (**Figure 9**).

**Figure 10:**
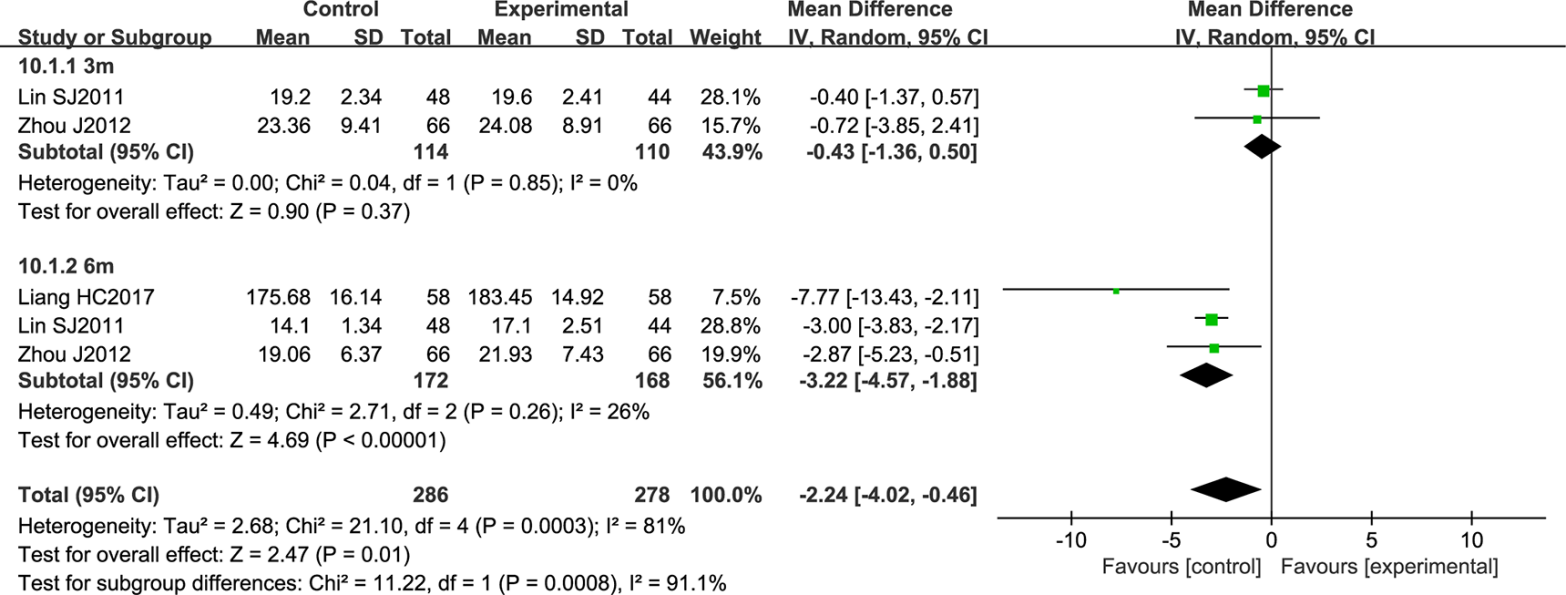
Forest plot of atherosclerotic plaque area in ischemic stroke patients treated with NSTC and conventional therapy (experimental group) and conventional therapy alone (control group).

Three studies comprising a total of 564 patients reported atherosclerotic plaque area. The data were analyzed by a random effects model according to the heterogeneity test (*P* = 0.0003; *I*2 = 81%). NSTC was shown to significantly reduce atherosclerotic plaque area (MD = -2.24; 95% CI [-4.02, -0.46]; *P* = 0.0008). After conducting a subgroup analysis according to the period of treatment, heterogeneity was strongly reduced in the two groups. Three months of NSTC treatment did not have a statistically significant effect (the diamond intersects the equivalent line) (**Figure 10**).

**Figure 11:**
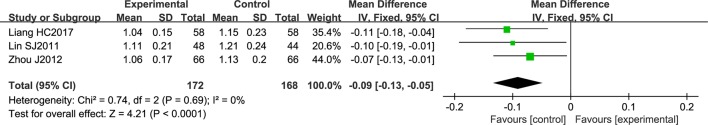
Forest plot of intima-media thickness in ischemic stroke patients treated with NSTC and conventional therapy (experimental group) and conventional therapy alone (control group).

NSTC significantly reduced intima-media thickness (IMT) (MD = -0.09; 95% CI [-0.13, -0.05]; *P* < 0.0001). There was no signiﬁcant heterogeneity noted among the individual studies (*P* = 0.63; *I^2^* = 0%; *P* = 0.69; *I^2^* = 0%), and a ﬁxed effect model was therefore employed for this analysis (**Figure 11**).

**Figure 12:**
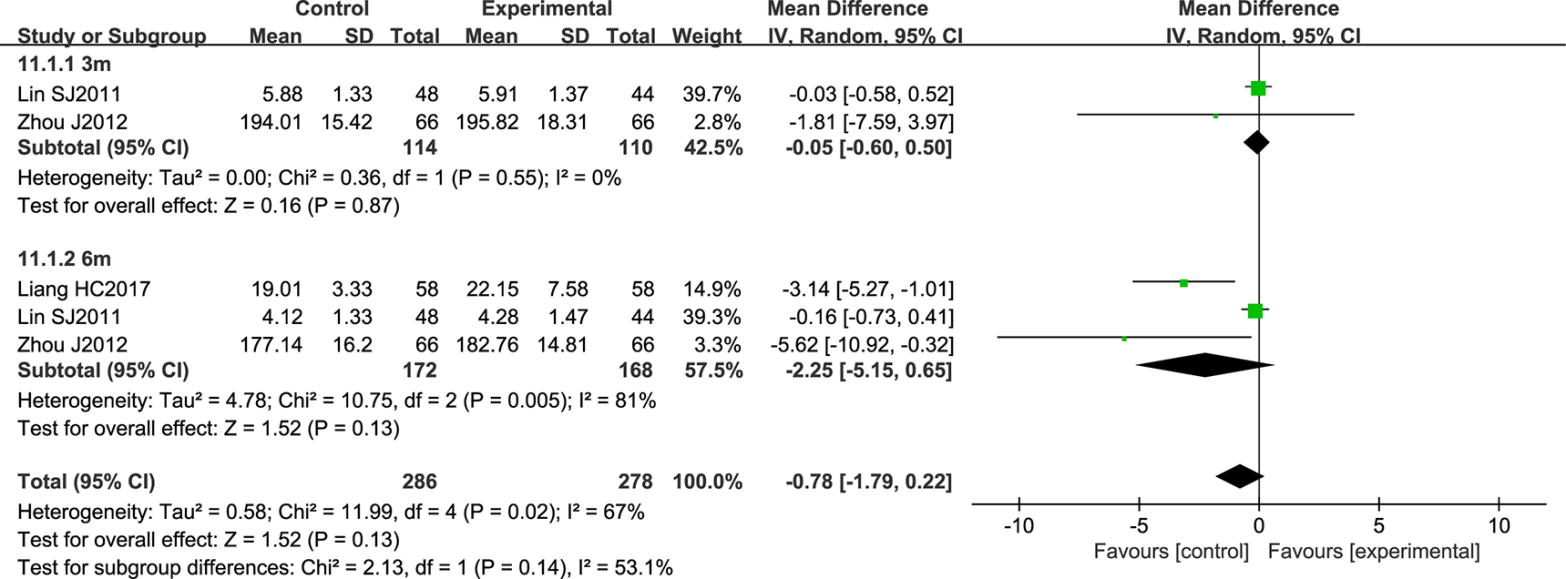
Forest plot of Crouse score in ischemic stroke patients treated with NSTC and conventional therapy (experimental group) and conventional therapy alone (control group).

NSTC did not have a statistically significant effect on the Crouse score (CS) (the diamond intersects the equivalent line). Here, the heterogeneity between the studies was substantial, and a random effects model was used (*P* = 0.02; *I^2^* = 67%) (**Figure 12**).

### Adverse Events

Adverse effects were reported in five trials. One trial reported gastrointestinal problems, and another two reported elevation of serum enzymes and occult blood in stool. None of the adverse effects noted were severe, and no life-threatening event was reported. No adverse effects of NSTC were identified in three trials.

### Publication Bias

Due to the small number of included trials (less than ten), it was impossible to conduct a sufficient additional analysis of publication bias.

## Discussion

Our review incorporated thirteen studies comprising a total of 1,360 participants. The aim of this meta-analysis was to provide an internationally accessible systematic review of the clinical efficacy and safety of NSTC for the treatment of IS. We found that NSTC may be an effective and safe treatment for IS. Additionally, we demonstrated that NSTC can preserve neurological function, decrease blood lipid level, and stabilize carotid atherosclerotic plaques.

The majority of patients who suffer a stroke survive the initial event, but may still face life-long functional disability (Cramer et al., 2017) due to the severe brain damage incurred (Langhorne et al., 2011). Stroke-related disabilities include deficits in mobility, cognition or communication, in addition to sensory impairment (Medical Rehabilitation Coordinating Committee, 2017). Thus, research into therapies that can improve the quality of life of patients in the chronic phase of stroke is critical. In this review, we explored the effect of NSTC on neurological function, employing a number of indexes for functional evaluation. The MBI was used to evaluate activities of daily living. The FMA was used to evaluate the effect of rehabilitation treatment. The FIM was used to evaluate cognitive and motor function. Finally, the ESS was used to evaluate neurological deficits. Our results showed that NSTC significantly improved neurological function in patients with acute ischemic stroke, while the efficacy of NSTC in convalescent patients was not statistically significant. It has been reported that the greatest part of recovery occurs in the first three months following stroke (Hatem et al., 2016). In this early stage, saving the ischemic region of dying neurons, promoting the restoration of damaged nerve function, and improving microvascular diastolic dysfunction may all promote recovery. Therefore, we assume that NSTC has greater efficacy when used in the acute phase of stroke.

Adiponectin is a 30-KDa protein hormone synthesized and secreted predominantly by white adipose tissue. It is involved in many processes related to atherosclerosis (Gairolla et al., 2017), such as lipid metabolism, inflammation, and endothelial dysfunction (Katsiki et al., 2017). Accumulating data has revealed that adiponectin levels favorably correlate with stroke risk, acting through antiatherogenic effects (Gorgui et al., 2017). Endothelial dysfunction is an early marker of atherosclerosis (Poredos and Jezovnik, 2013). Adiponectin exerts salutary effects by acting directly on vascular component cells including endothelial and smooth muscle cells, preventing neointimal proliferation and atherosclerosis (Zhang et al., 2016; Fujishima et al., 2017). Adiponectin levels have also been inversely associated with carotid IMT (Bevan et al., 2011; Gardener et al., 2012), and it is known that adiponectin can maintain endothelial function by enhancing the activity of endothelial nitric oxide synthase (Margaritis et al., 2013). Of the five ingredients contained in NSTC, Radix Paeoniae Rubra and Rhizoma Gastrodiae are known to decrease blood lipid level and stabilize atherosclerotic plaques (Xu et al., 2007; Lee et al., 2012; Shi et al., 2012). This may therefore explain the mechanisms of action by which NSTC exhibits efficacy in the treatment of IS. In this study, we found that NSTC can increase adiponectin level, reduce atherosclerotic plaque area, and decrease IMT. Taken together, this suggests that NSTC protects endothelial cells and exerts antiatherogenic effects, ultimately resulting in a beneficial effect in the treatment of IS.

From our analysis, NSTC seems a safe treatment, though it is difficult to make a conclusion with respect to safety given that only 30.8% of studies mentioned adverse events. The main side effects noted as result of NSTC treatment were not life-threatening and included gastrointestinal problems, elevation of serum enzymes, and occult blood in stool. However, some RCTs did not report adverse events, and none of the trials conducted statistical analysis of adverse events between the experimental and control groups. Through our comprehensive analysis of the existing evidence, we therefore concluded that the rate of adverse events as a result of NSTC treatment is relatively low.

The methodological quality of most of the included trials comprising small sample sizes was poor, and this may have reduced the reliability of our statistical analysis. Most trials did not mention random sequence generation, allocation concealment, blinding of participants and personnel, or blinding of outcome assessments. Although the intervention and control groups were subject to strict inclusion criteria, significant clinical heterogeneity existed in this meta-analysis. We assumed that variations in disease stage and basic condition may be important factors underlying this clinical heterogeneity, so more comprehensive and convincing scales are required in future studies. Overall, we assumed that the quality of the studies included in this meta-analysis was poor. **Figures 2** and **3** (summaries of our risk of bias assessment) demonstrate that many of the included studies had various inconsistencies. However, the data integrity and selective reporting of the studies were relatively objective and unbiased.

The observation period may have contributed to the significant heterogeneity observed in our study. After performing a subgroup analysis using different treatment periods, it was noted that heterogeneity greatly reduced. With respect to the FIM and FMA, due to the insufficient number of included studies, we did not conduct the funnel plot asymmetry test or subgroup analysis. When unexplained heterogeneity was noted, we utilized a random effects model. Moreover, although all the included studies reported comparable baseline characteristics between the experimental and control groups, the baseline values differed between the studies. We therefore could not exclude this bias, and this may also have contributed to the heterogeneity observed.

Finally, given that eligible studies published in other languages may have been excluded from this meta-analysis because we included only studies published in English and Chinese, publication bias cannot be excluded.

## Conclusion

The findings of this meta-analysis demonstrated that the use of NSTC in the treatment of IS can improve the overall response rate and neurological function, increase blood adiponectin level, reduce neurological deficits, and decrease atherosclerotic plaque area. However, owing to the small sample size and defective methodology of the included studies, these promising findings should be interpreted with caution. Therefore, future studies with a greater sample size and improved methodological quality are required to strengthen this existing evidence.

## Author Contributions

YiG and YoG developed the concept of the study. YX, JC, HS, and HZ developed and conducted the search strategy. LW and NA independently screened the titles and abstracts of all retrieved records. MY and CT conducted data extraction. HZ and XY performed meta-analysis. HZ wrote the draft of the manuscript. All authors read and approved the final version of the manuscript.
