# Supplementary material for: Efficacy and Safety of NaoShuanTong Capsule in the Treatment of Ischemic Stroke: A Meta-Analysis
Source: Front Pharmacol. 2019 Oct 11;10:1133. doi: 10.3389/fphar.2019.01133 (PMC6797837; doi:10.3389/fphar.2019.01133)
Supplement: Supplementary file 1 [file Image_1.pdf]

Supplementary Figure 1:

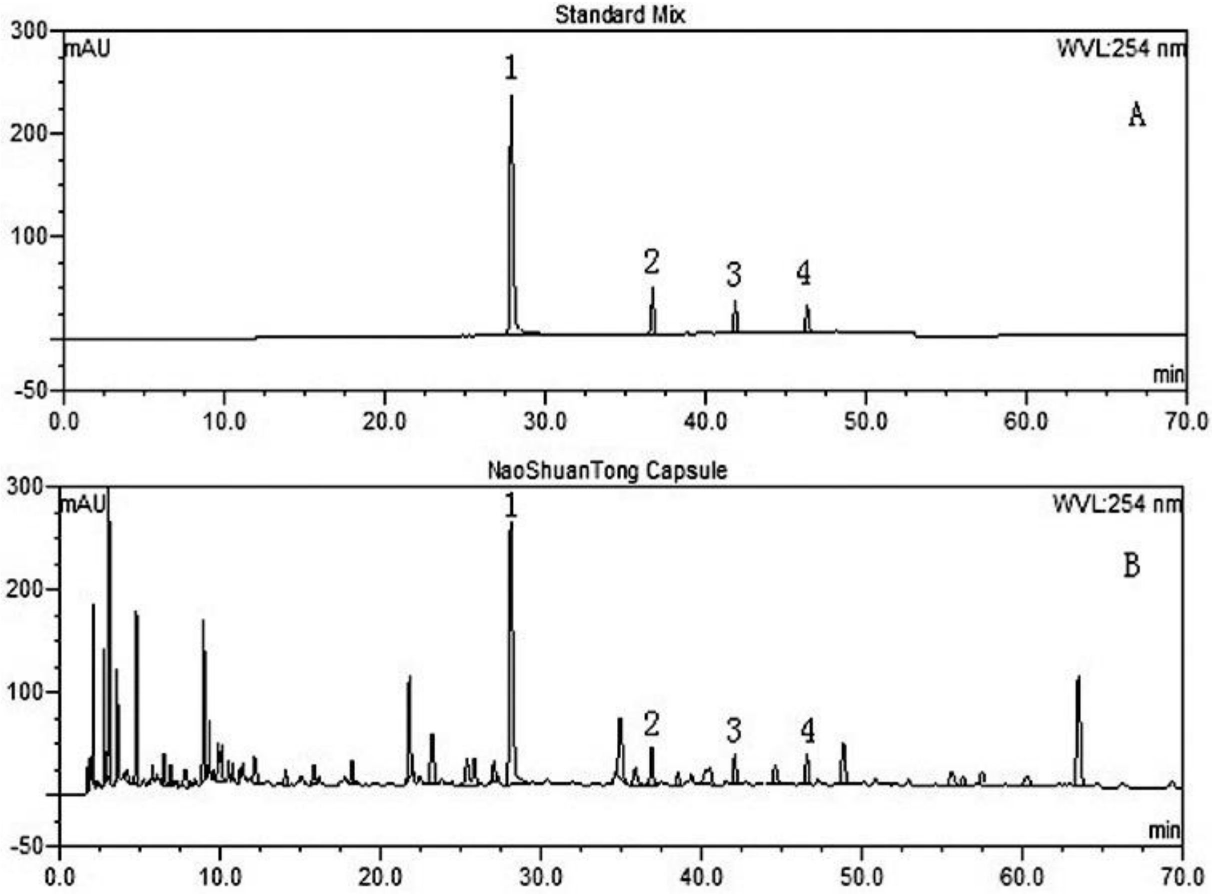

High performance liquid chromatography (HPLC) of standard mix (A) and NaoShuanTong capsule (B) using ultraviolet absorbance detection at wavelength 254 nm. 1: Paeoniflorin; 2: Ecdysterone; 3: Typhaneoside; 4: Isorhamnetin-3-O-neohesperidoside(Liu et al., 2014).

Liu, H., Peng, Y.Y., Liang, F.Y., Chen, S., Li, P.B., Peng, W., Liu, Z.Z., Xie, C.S., Long, C.F., and Su, W.W. (2014). Protective effects of traditional Chinese medicine formula NaoShuanTong capsule on haemorheology and cerebral energy metabolism disorders in rats with blood stasis. *Biotechnol Biotechnol Equip* 28, 140-146.
